# Early Detection of Cognitive Decline: Executive Functioning Index R4Alz-pc Predicts Memory-Related Concerns in Community Adults

**DOI:** 10.3390/diagnostics15172158

**Published:** 2025-08-26

**Authors:** Eudokia Emmanouilidou, Despina Moraitou, Nikoleta Frantzi, Eleni Poptsi, Emmanouil Tsardoulias, Andreas L. Symeonidis, Georgia Papantoniou, Maria Sofologi, Elvira Masoura, Glykeria Tsentidou, Ioanna-Giannoula Katsouri, Magda Tsolaki

**Affiliations:** 1 Laboratory of Psychology, Department of Cognition, Brain and Behavior, School of Psychology, Aristotle University of Thessaloniki (AUTh), 54124 Thessaloniki, Greece; despinamorait@gmail.com (D.M.); nkfrantzi@gmail.com (N.F.); emasoura@psy.auth.gr (E.M.); gltsentidou@gmail.com (G.T.); 2 Laboratory of Neurodegenerative Diseases, Center for Interdisciplinary Research and Innovation, Aristotle University of Thessaloniki (CIRI—AUTh), 54124 Thessaloniki, Greece; poptsielena@gmail.com; 3 Greek Association of Alzheimer’s Disease and Related Disorders (GAADRD), Petrou Sindika 13 Str., 54643 Thessaloniki, Greece; 4 School of Electrical and Computer Engineering, Faculty of Engineering, Aristotle University of Thessaloniki (AUTh), 54124 Thessaloniki, Greece; etsardou@ece.auth.gr (E.T.); symeonid@ece.auth.gr (A.L.S.); 5 Laboratory of Psychology, Department of Early Childhood Education, School of Education, University of Ioannina, 45110 Ioannina, Greece; gpapanto@uoi.gr (G.P.); m.sofologi@uoi.gr (M.S.); 6 Department of Occupational Therapy, Faculty of Health and Caring Sciences, University of West Attica, 12243 Athens, Greece; ykatsouri@uniwa.gr

**Keywords:** affect about memory, Alzheimer’s disease (AD), executive functions, memory concerns, memory worry, mild cognitive impairment (MCI), preclinical Alzheimer’s disease (pcAD), subjective cognitive decline (SCD)

## Abstract

**Background/Objectives:** Early identification of preclinical Alzheimer’s disease (AD) is critically important. This study investigates whether early cognitive decline in preclinical AD can be detected through the executive functioning index R4Alz-pc by predicting memory-related worry and negative affect. **Methods:** A sample of 105 cognitively healthy, community-dwelling older adults (M = 59.25 years) completed the Multifactorial Memory Questionnaire (MMQ) and the R4Alz-pc index battery. **Results:** Lower performance on the R4Alz-pc index was significantly associated with increased negative affect related to memory. Mediation analysis indicated that memory-related worry mediated the relationship between executive dysfunction and negative affect. **Conclusions:** This study highlights the importance of early, objective cognitive assessment using easy-to-administer and non-invasive tools like the R4Alz-pc, enabling timely detection and intervention for preclinical AD. Such methods may reduce reliance on costly biomarker-based diagnostics.

## 1. Introduction

Within the broader aim of identifying preclinical Alzheimer’s cognitive impairment in its earliest stage, Subjective Cognitive Decline (“SCD”) represents a particularly salient area of research interest [1,2,3,4,5]. It describes the self-experienced sense of cognitive decline reported by the individual, in the absence of objective impairment [6,7,8,9,10]. In other words, self-perceived memory concerns and cognitive complaints are typically classified under the concept of SCD in the relevant literature [11,12]. The decline reported is not always observable by others and frequently fails to be objectively validated by conventional neuropsychological measures, especially those developed for diagnosing MCI or dementia. A growing body of research in recent years suggests that SCD serves as the earliest predictive stage of Alzheimer’s disease (“AD”) pathology development [10,12,13,14]. Detection of SCD may be crucial for implementing interventions aimed at delaying the emergence of dementia or even reversing the cognitive decline in the very beginning [9]. Evidence shows that SCD may be present as early as 8 to 10 years before the diagnosis of AD dementia [9,10]. Furthermore, SCD appears to be a stronger predictor of dementia, when accompanied by individuals’ concerns about their memory performance [10]. It has been shown that individuals with SCD who progress to AD already exhibit amyloid-β in the brain from the early stages of cognitive impairment [15].

Due to its documented diagnostic value, SCD has recently attracted considerable research interest. Jessen and colleagues [4] suggest that its diagnosis relies on two main criteria. Specifically, the diagnostic framework requires (1) subjective and persistent complaints of cognitive decline relative to previous cognitive status, and (2) typical performance on standardized, demographically adjusted neuropsychological tests typically used for identifying MCI [10,16]. However, at the clinical level, SCD currently remains outside the scope of commonly used diagnostic categories [10], and accordingly, individuals with SCD are often categorized as cognitively normal, as they perform within normal limits on standard neuropsychological instruments [14].

Recently, the notion that individuals with SCD are cognitively healthy has been increasingly challenged by new findings, which suggest that these individuals may indeed exhibit cognitive decline [14]. These self-reported concerns, reflecting functional cognitive processes based on metacognitive skills, should be considered in clinical assessment rather than dismissed as mere emotional discomfort [11,12,17,18]. Individuals exercise metacognitive monitoring, meaning they possess the capacity to oversee and evaluate their own cognitive system. Therefore, they seem to be aware of their cognitive impairments, prompting their subjective complaints [19].

Recently, there has been increased literature interest in metacognition in relation to the preclinical stages of AD. To be more precise, metacognition is defined as the monitoring and evaluation of one’s cognitive abilities [19,20,21]. Even individuals with SCD or MCI can recognize their cognitive abilities, but awareness of deficits often leads to excessive worry and negative affect [22]. Memory complaints partly reflect age-related declines in cognitive control and processing speed [16], alongside heightened concern and dissatisfaction with memory performance [19].

Worrying about memory is one of the metacognitive elements of special interest that seems to be crucial for identifying SCD. In fact, worries about memory are considered a significant marker of SCD connected to neurodegenerative pathology [23]. Additionally, they have been associated with reduced performance in daily living activities in cognitively healthy people [24] and with neurobiological markers in brain regions relevant to memory function [25].

In addition to worries about memory function, the individual’s affective state and emotions concerning memory performance represents an important aspect in the evaluation of SCD. According to the Self-Regulatory Executive Function model [26], certain metacognitive beliefs contribute to the development of prolonged negative thinking, such as worry and the emergence of negative affect. Individuals who perceive their difficulties experience worry due to this negative thinking.

These psychological qualities are also supported neurobiologically, with evidence showing that changes in brain regions like the prefrontal cortex and hippocampus—key areas for memory and executive functioning—correlate with emotional reactions concerning memory [23]. It appears that affect about memory function serves as an indicator of cognitive decline rather than merely an emotional factor [23]. That is, both concerns about memory and the affective response to them represent metacognitive markers of SCD.

The concerns and cognitive complaints of individuals with SCD appear to primarily involve subjective memory ability. They develop negative affect regarding memory performance, as they primarily perceive cognitive decline as a memory difficulty. In many cases, there is also a decline in memory function [12,27]. However, in most cases, executive dysfunction seems to be the primary impairment within this diagnostic group [28,29]. Individuals may not directly perceive this decline, which is also challenging to identify with common neuropsychological measures assessing MCI and dementia. This might underlie the reason—given their performance in the existing tests—they are labeled as cognitively normal.

Until recently, the prevailing scientific view held that memory decline, especially episodic memory, was the first clinical manifestation of AD. However, emerging research suggests that executive functions may be disrupted at earlier stages of neurodegeneration, even before the clinical onset of the disease, as seen in cases of MCI and SCD [30,31,32]. For example, evidence from a large-scale cohort study indicated that individuals with SCD exhibit reduced performance in executive control measures. Specifically, impairments in cognitive flexibility and task-switching emerged as a distinctive deficit, implying that early cognitive dysfunction in SCD may be primarily reflected in executive subsystems before any measurable memory function impairment becomes apparent [33]. At the neurobiological level, age-related dysregulation of the dopaminergic system—particularly in projections to the prefrontal cortex—has been shown to adversely affect key aspects of executive functioning, including control of attention, working memory, and inhibitory control [34]. Moreover, a growing body of research, encompassing both biomarker evidence and neuropsychological assessments, supports the notion that executive dysfunction in SCD precedes memory decline [31,35]. These early disruptions appear to be associated with structural and functional alterations in frontal and prefrontal cortical regions, brain areas critically involved in cognitive control processes [36,37,38,39,40]. Timely identification of subtle impairments in higher-order cognitive processes with advancing age is therefore of critical importance [41,42,43,44], as it may enable the prevention or delay of the early neurodegenerative symptoms that typically lead to dementia, particularly that of the Alzheimer’s type [45].

Hence, based on the literature, the development of a screening instrument objectively distinguishing the preclinical stage of AD in its entirety would significantly enhance early detection efforts. Such a tool could allow for broad population-level screening beginning in midlife, facilitating earlier recognition of neurodegeneration-related cognitive deficits.

### The Purpose and the Hypotheses of the Study

The present study aims to examine whether cognitive decline related to preclinical (pc) AD can be detected from the very early stages, by a short and easy-to-administer test of executive functioning. Specifically, this study aims to examine whether worrying about memory and the emergence of negative affect related to memory can be predicted by an index of objective cognitive performance, particularly the executive functioning index R4Alz-pc.

Two research hypotheses were formulated based on this framework:

**Hypothesis 1.** 
*Higher concern about memory, expressed through elevated levels of worry and negative affect, will be associated with lower performance in the R4Alz-pc index.*

**Hypothesis 2.** 
*Subjective cognitive concerns about memory will be significantly linked to objective deficits in the main executive functions.*

## 2. Materials and Methods

### 2.1. Participants

A snowball sampling technique was utilized for participant recruitment. The sample was drawn from Macedonia and Thrace, North Greece, through announcements made on social media platforms. The primary inclusion criterion was age, with participants required to be between 45 and 74 years old (mean age = 59.25, SD = 5.21). As regards the exclusion criteria, participants had to be community-dwelling adults, free from any diagnosed neurological or psychiatric disease, as verified through self-reported screening questions.

All participants were of Greek ethnic origin. In terms of educational background, all participants reported having completed at least 13 years of formal education, indicating a relatively high level of educational attainment. No participant had been assessed previously for preclinical Alzheimer’s pathology. Thus, undetected cases of MCI or SCD may have been present.

The required sample size was found to be 68 participants, determined using the Linear Multiple Regression Random Model in G power analysis. The statistical power effect was set at 0.80, at a significance threshold of α = 0.05. However, a larger sample of over 100 participants was used in this study to enable the implementation of mediation analysis in a structural equation modeling (SEM) context. Thus, the sample consisted of 105 adults, including 35 men (33.3%) and 70 women (66.7%).

### 2.2. Tools

#### 2.2.1. R4Alz-pc Index

The REMEDES for the Alzheimer (R4Alz) battery, developed by Poptsi et al. [29,46], is a neuropsychological tool specifically designed to assess cognitive control, with the primary aim of distinguishing between cognitively healthy older adults, individuals with Subjective Cognitive Decline (SCD), and those diagnosed with Mild Cognitive Impairment (MCI) [42]. The battery is tailored to evaluate main cognitive control functions and has been shown to be a reliable and valid instrument for detecting subtle cognitive deficits, particularly in the early stages of cognitive decline, when such deficits are potentially reversible [29,42,44]. It includes assessments of critical cognitive domains such as working memory, attentional control system, inhibitory control, and cognitive flexibility. The tool has been validated and is free from the influences of educational level, gender, as well as psychological and other factors.

For the purposes of the present study, a part of the R4Alz battery, the R4Alz-pc (preclinical) index, was utilized. This specific configuration was adopted based on prior validation by Poptsi et al. [29], who demonstrated the index’s effectiveness in differentiating cognitively healthy older adults from those exhibiting SCD.

The R4Alz-pc index is a computerized tool conducted via a computer platform. It integrates multimodal stimuli—visual, auditory, and motor—to establish a dynamic and interactive assessment framework. Specifically, the system utilizes diverse modalities. Visual stimuli consist of animal sketches (cat, dog, and sheep), which participants are required to identify or react to (e.g., naming the animal depicted in the sketch upon illumination of a light cue). Auditory stimuli involve the sounds related to the aforementioned animals, serving either as confirmatory cues or as elements necessitating cognitive engagement (e.g., pressing the Enter button on the computer keyboard whenever the sound of a dog is heard). Additionally, light indicators, such as green or red, also function as feedback signals, offering information on whether the response was correct or incorrect. This multimodal approach facilitates comprehensive cognitive and sensorimotor evaluation in a controlled and reproducible environment. Prior to the commencement of each task, participants receive the corresponding instructions along with a non-evaluated example when deemed necessary.

The tasks that constitute the index are the following:

Inhibition task: It assesses the ability to inhibit automatic responses and consists of four conditions. In the first (A), participants name animal drawings; in the second (B), they identify animal sounds. In the third condition (C), participants are instructed to name the visual stimulus and ignore the auditory cue. Lastly, in the fourth condition (D), they must name the sound while ignoring the visual image. Scores range from 0 (optimal performance, no errors) to 60 (maximum errors).

Switching task: It evaluates the ability to shift between rules or tasks. In the first condition (A), participants name animal sounds until a red light prompts them to switch and name the corresponding visual stimulus instead. In the second condition (B), they must continuously alternate based on cues: white light indicates sound naming, red light indicates image naming. Scores range from 0 (best performance) to 63 (worst performance).

Cognitive Flexibility task: The first condition (A) evaluates the participant’s ability to combine inhibition and rule/task-switching, thus indexing cognitive flexibility. It comprises four sub-conditions requiring participants to deactivate the lights using the Enter button on the computer keyboard, based on simultaneous visual and auditory stimuli. The task involves sudden, unannounced shifts in the response rule, requiring participants to rely on performance feedback to identify the change and flexibly adjust their response strategy. For instance, in the initial condition, the participant is required to press the Enter key each time the light appears on the sketch of the dog, while both visual cues and animal sounds are presented concurrently for various animals. In the next condition, the response rule shifts, and the participant must now respond only to the auditory stimulus, specifically when the dog’s sound is played. Scores range from 0 (no errors) to 56 (maximum errors). The second condition (B) further assesses combined executive functions through four progressively challenging subtasks. Condition B involves two steps: (1) deactivating green and red lights from left to right in alternating color sequence, skipping repeated colors, starting with green; (2) performing the same task in reverse order (right to left), starting with red. Scores range from 0 (all correct) to 8 (one error per block).

The index is derived by aggregating the individual scores as given above. All variables were normalized within the range [0, 1] using min–max normalization. To compute the overall score for the index, the sum of the squared values was applied as follows:

$$S_{Executive\;Functioning}={X_{ICT-RST\;1\&2}^{\prime }}^{2}+{X_{CFT}^{\prime }}^{2}+{X_{CFT\;cond\;b}^{\prime }}^{2}$$

Squaring the values amplifies the differences between high and low scores, assigning greater weight to higher performances (i.e., lower error values), while diminishing the influence of lower scores. Each of the variables contributes equally to the total score, which theoretically ranges from 0 (optimal performance) to 3, as the min–max normalization bounds each score to the [0, 1] range.

The composite score of the R4Alz-pc demonstrated high diagnostic accuracy in the initial study of its validation, with an extremely high sensitivity of 91.3% and very high specificity of 82.4% in distinguishing between cognitively healthy older adults and individuals with SCD, with a cut-off score of 0.401 [29]. The R4Alz-pc index has been validated in terms of its structural validity and convergent validity, through comparison with a series of well-established neuropsychological instruments. Furthermore, its sensitivity and specificity have been demonstrated in effectively distinguishing between healthy aging and different stages of preclinical AD [29,42,44,46].

#### 2.2.2. Multifactorial Memory Questionnaire (MMQ)—Satisfaction with Memory Functioning Subscale

The Multifactorial Memory Questionnaire (MMQ), developed by Troyer and Rich [47], is a self-report instrument consisting of 57 items aimed at clinicians and researchers for the assessment of metacognition in middle-aged and older adults. It includes three subscales: The Satisfaction with Memory Functioning (MMQ How I Feel About My Memory Scale), the Self-Perceived Memory Ability (MMQ Memory Mistakes Scale), and the Self-Reported Use of Memory Strategies (MMQ Use of Memory Strategies Scale). For this study, we utilized the translated version of the first subscale (Satisfaction with Memory Functioning) into Greek, as adapted by Emmanouilidou et al. [48].

The “How I Feel About My Memory” subscale (Satisfaction with Memory Functioning) contains 18 statements that reflect an individual’s emotional responses and concerns regarding their current memory condition. Participants respond on a five-point Likert scale (from 1 = strongly agree to 5 = strongly disagree), according to their level of agreement. The item scores are summed to two separate dimensions (based on the factors that structure the questionnaire), with higher scores indicating more concerns and more negative emotions related to memory functioning, respectively. It includes items like “I feel unhappy when I think about my memory ability” and “I worry that I will forget something important”.

Regarding the structural validity and reliability of the scale in its Greek version, two factors emerged from the application of Exploratory Factor Analysis to data in Greek [48]. The first factor reflects an individual’s concerns about their memory, while the second represents the affect the individual has regarding their memory. Internal consistency reliability was calculated using Cronbach’s alpha, which yielded values of α = 0.915 for the first factor (worry about memory) and α = 0.916 for the second factor (affect about memory). The Greek version of the Multifactorial Memory Questionnaire (MMQ) was used under license from *Baycrest* Centre for Geriatric Care, Toronto.

### 2.3. Procedure and Ethical Considerations

Data were collected through individual, in-person assessments with participants. A total of 105 adults took part in this study over a three-month period, from January to March 2025. The assessment was conducted in a quiet environment with a comfortable temperature, either at the residence of one of the researchers or at the participant’s home or professional office. The total administration time for the tasks ranged from 50 min to 1 h. The execution of the R4Alz-pc index lasted approximately 20 min. The examiner explained the procedure based on the official electronic guidelines of the index. Supplementary explanations were offered when necessary to ensure participant understanding. In cases where the task contained an illustrative example, it was shown to the participant unless they opted out.

All participants were provided with a written information sheet detailing the aims of this study, the voluntary nature of participation, and the confidentiality of their personal data. Compliance with the General Data Protection Regulation (GDPR) of the European Union, in effect from 28 May 2018, was ensured. Informed consent was obtained in writing prior to data collection, in accordance with the ethical principles outlined in the Declaration of Helsinki (World Medical Association, 2013). The study protocol was reviewed and approved by the Ethics Committee of the Alzheimer Hellas organization, in accordance with national and international standards for research involving human participants (number of decision protocol code 94, approved on 20 December 2023).

Individual demographic data were first obtained. This was followed by the administration of the Multifactorial Memory Questionnaire—Satisfaction with Memory Functioning and the R4Alz-pc index. The order of administration of the main instruments was counterbalanced to minimize potential order effects.

### 2.4. Statistical Analysis

Data analysis was conducted using the Statistical Package for the Social Sciences (SPSS) v.29.0.1.0 and the JASP v.0.19.3.0. Initially, descriptive statistics were computed—specifically, the mean (M) and standard deviation (SD) for all the 3 variables, that is, worry about memory, affect about memory, and R4Alz-pc. Pearson’s correlations were computed to clarify the relationships among memory worries, affect about memory, and objective cognitive performance (R4Alz-pc score). Subsequently, mediation analysis was performed to examine both the direct and indirect effects of the R4Alz-pc performance (objective measurement) on affect about memory, with memory worries functioning as the mediating variable.

Mediation analysis serves to elucidate the underlying mechanisms through which a predictor variable exerts its influence on an outcome variable. Specifically, it posits that the observed association between the predictor and the outcome may be partially or fully transmitted through a third variable, termed the mediator. This analytical framework allows for the distinction between direct effects—the influence of the predictor on the outcome independent of the mediator—and indirect effects, which capture the portion of the relationship that is channeled through the mediator. In the present study, mediation analysis was conducted within the framework of structural equation modeling (SEM) using path analysis models incorporating mediating variables, implemented in JASP version 19.

## 3. Results

Descriptive statistics (mean and standard deviation) were calculated for the three variables of interest in the study (see Table 1).

Analysis of the frequency of performance above and below the cut-off score of 0.401 [29] in R4Alz-pc revealed that 96 out of 105 participants (91% of the total sample) exhibited objective cognitive control deficits at the level of core executive functions. Hence, only nine people were cognitively healthy, according to this index (see Table 2).

A statistically significant and high positive correlation was found between worry about memory and affect about memory. Significant positive correlations were also found between worry about memory, affect about memory, and R4Alz-pc performance. These findings indicate that, among individuals aged 45–72, greater concern about memory is associated with more negative emotions regarding memory, and both with a higher number of errors on the objective cognitive control measure. This pattern suggests an underlying slow decline in executive functioning within this age range, which is associated with the subjective estimations and feelings about memory that people experience and express.

Correlation analyses indicated a strong positive association between worry about memory and affect about memory, *r*(n − 2) = 0.79, *p* < 0.01. Worry about memory was also moderately positively associated with the R4Alz-pcAD scale, *r*(n − 2) = 0.26, *p* < 0.01. Furthermore, affect about memory was positively associated with the R4Alz-pcAD scale, *r*(n − 2) = 0.32, *p* < 0.01.

To further investigate the relationships among objective cognitive control performance, worry about memory, and affect about memory, we conducted a mediation analysis (see Table 3). It is important to note that, given the cross-sectional design of this study, only directional relationships can be depicted, rather than causal effects. R4Alz-pc was set as the predictor variable (input variable), worry about memory as the mediator, and affect about memory as the outcome variable (dependent variable) (see Table 4).

The analyses revealed a statistically significant indirect effect of objective cognitive control performance (R4Alz-pc) on affect about memory, mediated by worry about memory. Specifically, individuals appeared to present a decline in their cognitive control performance, which led to increased worry about their memory. This worry, in turn, contributed to more negative affective evaluations of their memory function. Thus, objective cognitive control performance, as more errors in executive functioning, was found to be significantly associated with increasing negative affect about memory. In other words, in this age range, worry and affect about memory appear to be grounded in measurable reductions in executive functioning.

## 4. Discussion

Cognitive complaints are a common occurrence among adults and constitute a main reason for referrals to, or visits at, memory clinics [49]. In the literature, such complaints are indicative of Subjective Cognitive Decline (SCD), as they reflect individuals’ perceptions of memory deterioration relative to their previous baseline cognitive functioning. It is also a diagnostic criterion for Mild Cognitive Impairment (MCI), a preclinical stage of AD that, however, can be detected by objective measures too [45]. The importance of this lies in its function as a potential prodromal marker of cognitive deficits in the context of pathological processes such as Alzheimer’s disease [10] or cardiovascular disorders [50]. At this stage, standard neuropsychological tools are insufficient for cognitive decline detection, as cognitive decline is very slow and tends to manifest predominantly in executive functions or cognitive control abilities rather than memory, which is the most common function that laypeople people can observe and estimate everyday [12,14,29].

A critical challenge encountered in both daily functioning and clinical settings relates to memory complaints reported by relatively young individuals in midlife. This raises the question of whether these complaints warrant consideration in clinical settings, and whether affected individuals may already experience genuine cognitive impairment at a stage when only subjective concerns are present and objective performance seems to remain intact. Such worries are often associated with negative affect about memory function [26]. Accordingly, the present study sought to assess whether worry about memory and negative affect—as core components of SCD and MCI [alternatively classified together as preclinical Alzheimer’s disease (pcAD)]—are related to objective markers of cognitive impairment, especially in complex cognitive functions—like executive functions or cognitive control abilities—that may decline in the earliest stages of neurodegeneration. The principal aim was to conduct a pilot study to demonstrate that it is indeed possible to establish an instrument with the potential to accurately detect cognitive decline from the very beginning of the preclinical continuum of AD. The findings of this study indicate that the R4Alz-pc index has the potential to detect cognitive impairment at its very early stage, as it captures core executive function deficits, one of the earliest domains to decline in the course of neurodegenerative progression.

During midlife and later in aging, cognitive health should coexist with physical well-being [51], characterized by the absence of cardiovascular and metabolic diseases (including diabetes and hypercholesterolemia), neurological and psychiatric disorders, autoimmune conditions, and cancer. Healthy aging is an attainable goal, yet it necessitates the adoption of protective lifestyle behaviors from an early age. These include avoidance of smoking, alcohol, and substance abuse; residence in environmentally clean and oxygen-rich settings; regular engagement in aerobic physical activity; and adherence to a nutritionally balanced diet. Among modifiable factors, nutrition has consistently emerged in longitudinal studies as a critical determinant of healthy aging. The Mediterranean and DASH (Dietary Approaches to Stop Hypertension) diets are most frequently highlighted for their protective role in both physical and cognitive health [52]. In the recent longitudinal study by Tessier et al., only 9.3% of the total sample reached the age of 70 in a healthy state [52]. This finding appears to be consistent with the present study, in which only 9% of participants were found to be cognitively healthy.

Emerging evidence indicates a strong association between physical and cognitive health [51]. The R4Alz-pc index has shown that more than half of the individuals in the pilot sample of the present study demonstrate slightly below optimal performance, suggesting early subtle cognitive inefficiencies. This finding supports the hypothesis that cognitive decline in midlife follows a recognizable pattern, primarily involving deficits in executive functions [29]. The presence of such early decline underscores the need for systematic cognitive screening in this population. Indices such as the R4Alz-pc index may serve as efficient screening tools, given their ease of use, time efficiency, and cost-effectiveness. When cognitive decline is detected—even at this very early stage—it should prompt a thorough diagnostic work-up to identify underlying causes and enable timely intervention, targeting both cognitive and physical pathologies. Early-stage cognitive impairment may be attributable to AD pathology, cardiovascular conditions such as hypertension, myocardial infarction, or small vessel disease [10,50,53], but also to other neurological disorders, including Multiple Sclerosis [54] and Parkinson’s Disease [55]. Depending on the identified etiology, appropriate therapeutic strategies should be implemented. These may range from antihypertensive treatment in the case of vascular pathology [56] to disease-modifying monoclonal antibodies targeting amyloid pathology in AD [57], as well as non-pharmacological interventions such as cognitive exercise [58], physical exercise, and dietary modifications [52].

At a more specific level, the main hypothesis of the present study was that increased memory-related concern, as reflected in heightened levels of worry and negative affect, is expected to be associated with lower performance on the R4Alz-pc index. This hypothesis was confirmed, as a statistically significant association was found between performance on the R4Alz-pc index and both worry and affect about memory. The greater the number of errors made on the R4Alz-pc, the more negative individuals’ affect toward their memory became, as mediated by increased levels of worry about memory. Metacognitive evaluation and affective responses toward memory emerge through a dynamic interaction between actual cognitive control performance and the individual’s interpretation of that performance.

The extant literature supports that higher levels of worry about memory predict poorer performance on challenging objective cognitive tasks, suggesting that this worry reflects underlying cognitive impairments rather than merely subjective concern. Negative affect, which correlates with SCD [19,59], appears to reflect impairments in cognitive control. Previous studies have shown that individuals’ global self-assessments of cognitive errors—not limited to memory lapses—are associated with negative feelings about memory, which in turn mediate poorer performance on the Feature Binding Test (Condition 1) [48]. Regarding worry about memory, Kessler et al. [60] report that approximately 50% of older adults express concern about their memory, and this worry has been associated with SCD and the early stage of objective cognitive impairment. From a metacognitive perspective, such negative affect is more likely to emerge in individuals who struggle to realistically and adaptively interpret their perceived cognitive decline. Furthermore, negative affect has been shown to predict lower performance on tasks involving working memory and executive functioning [61]. While individuals appear to recognize that they experience cognitive difficulties relative to their previous functioning, most complaints focus on memory, as these difficulties are commonly and often wrongly attributed to a decline in memory capacity [12,19,28].

Regarding the second hypothesis of this study—that subjective cognitive concerns about memory will be significantly linked to objective deficits in major executive functions—subjective cognitive complaints are commonly used as self-reported perceptions of cognitive performance and serve as a screening tool for identifying SCD. These complaints are most frequently related to memory, with individuals often reporting forgetfulness or difficulty recalling information. However, many individuals fail to recognize that their cognitive difficulties might be rooted in executive dysfunctions [62].

At this stage, executive function impairment is typically subtle and may go unnoticed by individuals, as it manifests in more complex cognitive domains that are not easily captured by standard neuropsychological tests. Consequently, the perception that “my memory isn’t working” may actually reflect underlying impairments in executive processes [63]. This is due to the involvement of executive function mechanisms in the encoding and retrieval of information from memory. Specifically, reduced or inadequate inhibitory control has been observed, impairing the individual’s ability to suppress competing information during recall, which consequently affects the accurate retrieval of the required information. Additionally, impairments are noted under dual-task conditions [64]. There is also a decline in selective and divided attention, leading to erroneous or failed encoding and subsequent memory retrieval failure [65]. Furthermore, diminished cognitive flexibility results in information loss during encoding and difficulty in recalling information necessary for the specific task. The ability to shift between strategies and tasks, which is critical for the accurate retrieval of relevant information, is reduced or impaired [66].

Webster-Cordero et al. [63] found that individuals with such complaints primarily showed deficits in attention, working memory, cognitive flexibility, planning, and decision-making. The theory of retrogenesis, which suggests that higher-order cognitive functions are the first to decline in neurodegenerative diseases, further supports the idea that impairments in executive functioning are often evident early, even before memory-related deficits become prominent [67]. This aligns with the present study, which investigates the utility of the R4Alz-pc index to detect early-stage cognitive decline [29]. The R4Alz-pc index focuses on core cognitive control functions that play a pivotal role in the processes of encoding, storing, and retrieving information from memory [29,42,44]. Its particularly strong association with worry and negative affect related to memory performance suggests that it may serve as a reliable indicator of subjective difficulties. Consequently, in cases where individuals report concerns or experience negative emotions regarding their memory abilities, administration of this tool is deemed appropriate in order to determine whether there is objectively verifiable impairment in cognitive control. If such impairment is confirmed, further investigation into its underlying causes becomes necessary.

To conclude, the relationship between subjective concerns about memory, affect regarding memory, and objective cognitive control performance is complex and interrelated. The correlational model proposed in the present study emphasizes that lower objective performance triggers greater worry about memory, and in this way enhances negative affect toward memory. Given these findings, this study underscores the importance of using psychometrically sound and objective tools, such as the R4Alz-pc index, to assess executive function impairment, as these tools have the potential to detect preclinical AD from its very beginnings.

### 4.1. Limitations of the Study

Despite the clinical relevance of these findings, the present study is not without limitations. To begin with, this study employed a relatively small sample, which might limit the generalizability of the findings to the broader population. Due to its cross-sectional design rather than a longitudinal one, this study does not permit causal interpretations of the results. The main limitation is the absence of biological markers (e.g., CSF) and neuroimaging data (MRI, PET) that could be correlated with our index, in order to elucidate the underlying causes of cognitive decline.

### 4.2. Study Strengths

A major strength of this study is the use of the R4Alz-pc index, a valid, reliable, and easy-to-administer neuropsychological screening tool capable of detecting even subtle declines in cognitive ability from midlife onward, well before the onset of overt clinical symptoms. This early detection capability is critical for identifying individuals at risk and guiding them toward timely preventive or therapeutic interventions. Its non-invasive, cost-effective, and accessible nature makes it suitable for large-scale population screening, offering a practical alternative to expensive and logistically challenging biomarker-based assessments, and supporting broader implementation in both clinical and community settings.

### 4.3. Future Research Recommendations

Future research should employ longitudinal designs to elucidate causal relationships among the variables. Particularly crucial is the integration of neuroimaging data, such as MRI and PET, together with biomarkers like amyloid-β and white matter hyperintensities (WMHs) [68], to support further exploration of the neurobiological basis of preclinical stages. Finally, cognitive interventions aimed at strengthening executive functions are recommended to enhance cognitive resilience and potentially reverse the impairment.

## 5. Conclusions

In summary, this study demonstrated the critical relevance of objectively assessing cognitive impairment in adults as early as possible, in the absence of manifest clinical symptomatology. The employment of valid, easy-to-administer neuropsychological assessment tools such as the R4Alz-pc index, as screening tools in the general population, seems necessary both for early symptom identification and for providing direction in designing interventions that can stabilize cognitive decline. Such tools offer a promising avenue for the identification of cognitive impairment related to AD pathology from its beginnings, potentially reducing reliance on biomarker-based assessments, which can be prohibitively expensive and logistically demanding.

## Figures and Tables

**Table 1:** Mean (M) and standard deviation (SD) of the objective cognitive control index and the subjective concerns and feelings about memory.

Variable	M	SD
worry about memory	18.733	8.001
affect about memory	11.723	5.398
R4Alz-pc	1.308	0.822

**Table 2:** Frequency of R4Alz-pc scores above the cut off.

Range of Composite Score of R4Alz-pc	Number of Participants
0.06–0.400	9
0.406–0.49	5
0.5–1	34
1.1–1.4	20
1.5–2	23
2.1–2.4	4
2.5–3	5
3.1–3.4	1
3.5–4	2
4.1–4.4	0
4.5+	1

**Table 3:** Direct Effects of the predictor variable on the outcome variable.

						95% Confidence Interval	
		Std. Estimate	Std. Error	z-Value	*p*	Lower	Uper
**R4Alz-pc →**	**AFFECT**	0.117	0.061	1.926	0.054	−0.002	0.237

**Table 4:** Indirect Effects of the predictor variable through the mediating variable on the outcome variable.

							95% Confidence Interval	
			Std. Estimate	Std. Error	z-Value	*p*	Lower	Uper
**R4Alz-pc →**	**WORRY →**	**AFFECT**	0.199	0.069	2.875	0.004	0.063	0.335

WORRY = worry about memory; AFFECT = affect about memory.

## Data Availability

The data are not publicly available due to privacy and ethical restrictions but are available from the corresponding author upon reasonable request.
